# Transcriptomic study to understand thermal adaptation in a high temperature-tolerant strain of *Pyropia haitanensis*

**DOI:** 10.1371/journal.pone.0195842

**Published:** 2018-04-25

**Authors:** Wenlei Wang, Fei Teng, Yinghui Lin, Dehua Ji, Yan Xu, Changsheng Chen, Chaotian Xie

**Affiliations:** 1 Fisheries College, Jimei University, Xiamen, China; 2 Key Laboratory of Healthy Mariculture for the East China Sea, Ministry of Agriculture, Xiamen, China; Jawaharlal Nehru University, INDIA

## Abstract

*Pyropia haitanensis*, a high-yield commercial seaweed in China, is currently undergoing increasing levels of high-temperature stress due to gradual global warming. The mechanisms of plant responses to high temperature stress vary with not only plant type but also the degree and duration of high temperature. To understand the mechanism underlying thermal tolerance in *P*. *haitanensis*, gene expression and regulation in response to short- and long-term temperature stresses (SHS and LHS) was investigated by performing genome-wide high-throughput transcriptomic sequencing for a high temperature tolerant strain (HTT). A total of 14,164 differential expression genes were identified to be high temperature-responsive in at least one time point by high-temperature treatment, representing 41.10% of the total number of unigenes. The present data indicated a decrease in the photosynthetic and energy metabolic rates in HTT to reduce unnecessary energy consumption, which in turn facilitated in the rapid establishment of acclimatory homeostasis in its transcriptome during SHS. On the other hand, an increase in energy consumption and antioxidant substance activity was observed with LHS, which apparently facilitates in the development of resistance against severe oxidative stress. Meanwhile, ubiquitin-mediated proteolysis, brassinosteroids, and heat shock proteins also play a vital role in HTT. The effects of SHS and LHS on the mechanism of HTT to resist heat stress were relatively different. The findings may facilitate further studies on gene discovery and the molecular mechanisms underlying high-temperature tolerance in *P*. *haitanensis*, as well as allow improvement of breeding schemes for high temperature-tolerant macroalgae that can resist global warming.

## Background

*Pyropia*/*Porphyra* (Bangiales, Rhodophyta) is one of the most economically important mariculture crops. Its annual harvest was more than 1,806,000 tons (fresh weight) in 2014 [1]. *Pyropia* aquaculture has become one of the biggest artificial marine ecological experiments. In China, the yield of *P*. *haitanensis* reached 75% of the total output of *Pyropia* [2], which provide a great contribution to the economic aggregate of Fujian and Zhejiang Provinces. The market demand for *P*. *haitanensis* has increased due to its wide range of applications in the production of food, medicines, fertilizers, and biofuels and potentially “blue carbon” species [3, 4].

The optimal temperature range for growing of *P*. *haitanensis* blades is 15°C to 26°C. However, *P*. *haitanensis* farms often undergo sustained high temperatures that are induced by ‘temperature rebound’ in autumn in South China [5, 6]. High-temperature stress has recently resulted in a severe reduction in *P*. *haitanensis* output by inhibiting survival of the conchospores, enhancing premature senility, as well as inducing germling disease and eventual decay [7]. Therefore, it is necessary to breed new thermotolerant strains for alleviating the severely detrimental impact of high-temperature stress on *Pyropia* production. Some thermotolerant strains of *P*. *haitanensis* have been successfully screened such as Z-61 and ZS-1. Their capacity to resist high temperature has been validated by field cultivation in South China [5, 7]. These strains have higher yield and better quality and are thus widely cultivated.

The elucidation of the molecular mechanisms underlying the persistence of plants to high temperature stress may facilitate in the development of novel breeding technologies to generate thermotolerant plants [8]. Furthermore, it has been demonstrated that *Pyropia* has high tolerance for various abiotic stressors and are thus considered as a research model for studying stress physiology in seaweed communities [3]. However, our understanding of the molecular changes influencing various biochemical and regulatory pathways relating to responses to high temperature stress in *P*. *haitanensis* strains is limited. Choi et al (2013) reported that several of the high temperature (25°C) response transcripts in *P*. *tenera* treated within 3 h do not match known genes in current public databases, suggesting the existence of a novel mechanism to resist high temperature [9]. Furthermore, the H_2_O_2_ contents, heat shock protein 70 mRNA levels, NADPH oxidase activity, and the floridoside content in the thalli of *P*. *haitanensis* significantly increased in the presence of high temperature (35°C for 30 min) [10]. Sun et al. (2015) revealed heat treatment (24°C for 6 h) induced metacaspases genes up-regulated in *P*. *yezoensis*, which regulate programmed cell death, thereby accelerating the formation of generative cells [11].However, these thallus used in the abovementioned study were not high temperature-tolerant strains (HTT), and most reports have typically focused on the short-term high temperature stress (SHS).

In our previous study, we found HTT of *P*. *haitanensis* involves a higher number of differentially expressed genes and more extensive fold-changes in transcripts abundance compared to the wild-type. HTT strains could adapt to 29°C, whereas the thalli of the wild-type strains had disintegrated at the same temperature within 24 h [12]. The mechanisms underlying the responses of plants to high temperature stress vary with plant type as well as degree and duration of exposure [13]. Therefore, in the present study, gene expression and regulation in response to a time series (0 h, 3 h, 6 h, 12 h, 24 h, 2 d, 4 d, and 6 d) of high temperature stresses were examined by performing genome-wide high-throughput transcriptomic sequencing of HTT strain in *P*. *haitanensis*. The findings of the present study may help us better understand the defense mechanisms behind HTT of *P*. *haitanensis* in response to sustained high temperature, as well as provide information that may be applicable to the genetic breeding of thermotolerant strains.

## Materials and methods

### Seaweed materials and high temperature treatments

*P*. *haitanensis* strain Z-61 [5], the thermotolerant plant material used in this study, was selected and purified by the Laboratory of Germplasm Improvements and Applications of *Pyropia* in Jimei University, Fujian Province, China. The blades of *P*. *haitanensis* were cultured in tanks with Provasoli’s enrichment solution (PES) medium at 21 ± 1°C under cool-white fluorescent illumination (50–60 μmol photons·m^-2^·s^-1^) in a 10 h:14 h light: dark (L:D) cycle. The cultured medium was refreshed every 2 days. For heat stress treatments, 15 ± 2 cm long blades were randomly selected and cultured in aerated flasks (500 mL) at 29°C (high temperature) for 0 h, 3 h, 6 h, 12 h, 24 h, 2 d, 4 d, and 6 d. The other culture conditions are as earlier described. Each treatment was conducted in two biological replicates from independent flasks and cultivated in parallel.

### RNA extraction, *de novo* transcriptome assembly, and annotation

Total RNA was isolated from each of the above listed samples according to by Ji et al. [14] using an E.Z.N.A. Plant RNA Kit (Omega, Germany). The quality and quantity of the purified RNA were determined by measuring the absorbance ratio at wavelengths of 260 nm and 280 nm (A260/A280) and 260 nm and 230 nm (A260/A230) using a Nanodrop ND-1000 spectrophotometer (LabTech, USA). Only RNA samples with an A260/A280 ratio between 1.9 and 2.1 and an A260/A230 ratio greater than 2.0 were used in the subsequent analyses. The integrity of the RNA samples was assessed by 1.2% agarose gel electrophoresis. *De novo* transcriptome assembly and annotation were completed by GENE DENOVO Biotech, Ltd. (Guangzhou, China) as described Xie et al. [15]. The quality of the assembly was assessed by mapping the Illumina reads to the assembled transcriptome.

### Principal component analysis (PCA)

To detect differences in expression patterns across samples, PCA was performed on all 16 transcriptomic datasets using the “fast.prcomp” function of R (http://www.r-project.org/) on centered, but unscaled data. The parameters “retx”,”scale”, and”center” were equal to “T”, “F”, and “T”, respectively.

### Differential expression analysis of unigenes

For normalization of the data, gene expression levels were measured by the number of uniquely mapped reads per kilobase of exon region per million mappable reads (RPKMs), which could eliminate the influence of different gene lengths and sequencing discrepancies on gene expression calculation [16]. The RPKMs ratio between different samples was converted to fold-changes in the expression of each unigene. The unigenes with a value of |log_2_ fold change| ≥ 1 and the false discovery rate ≤ 0.001 were considered significantly differentially expressed genes (DEGs). We compared the gene expression level at high temperature/time points with control temperature (21°C/0 h). All DEGs were annotated with the NR, KEGG, GO, and other databases.

### Real-time quantitative PCR (qRT-PCR) verification

The relative expression levels of 12 randomly selected DEGs detected by RNA-Seq technology under different heat conditions were verified by qPCR. The ubiquitin-conjugating enzyme (*PhUBC*) gene was used as internal control [17]. qRT-PCR was run in triplicate for each sample. The information on the primers used for qPCR analysis is listed in S1 Table. qRT-PCR analysis was performed as described by Ji et al. [14].

## Results

### *De novo* assembly and functional annotation

**Fig 1:**
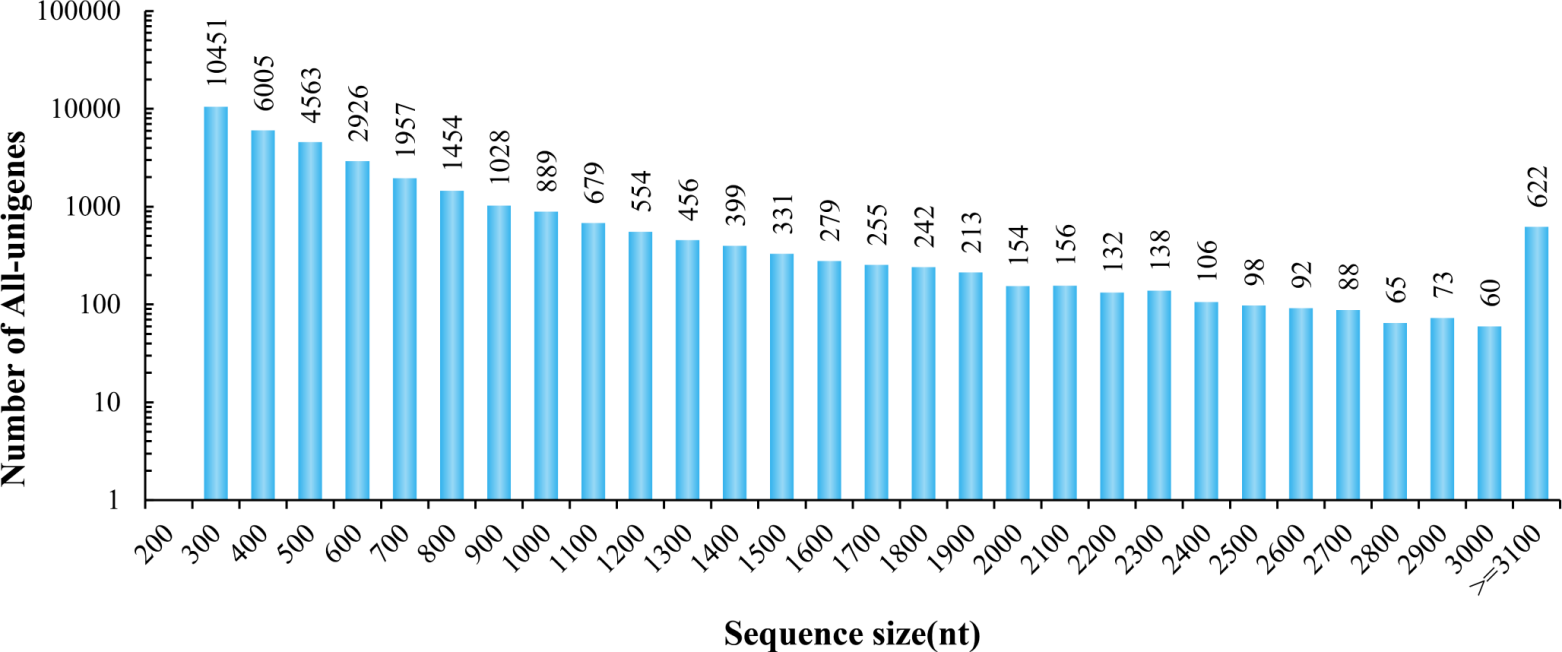
Length distribution of all unigenes mapped from contigs of the *P*. *haitanensis* transcriptome.

A total of 16 RNA samples generated from two biological replicates of *P*. *haitanensis* subjected to eight heat stress treatments were subjected to RNA-Seq. By Illumina high-throughput sequencing, a total of 642,661,468 high-quality nucleotide (nt) paired-end reads were obtained from the 16 libraries with 94.96% Q_20_ bases after the quality filtering step (Table 1). The percentage of uniquely mapped reads was approximately 70% by mapping the Illumina reads to the assembled transcriptome (S1 File). The raw data generated here were deposited to the NCBI Short Read Archive (SRA) database as accession number PRJNA428906. These high-quality short reads were assembled into 34,465 unigenes using the Trinity *de novo* assembly program. The length of these assembled unigenes with 66.14% GC percentage ranged from 201 bp to 18,142 bp (Fig 1).

**Table 1:** Summary of the *P*. *haitanensis* transcriptome.

Item	Number
**Total number of clean reads**	642,661,468
**Total base pairs (bp)**	80,332,683,500
**Average read length**	125
**Q_20_**	94.96%
**GC percentage**	66.14%
**Total number of unigenes**	34,465
**Average length (bp)**	646.60
**N_50_ (bp)**	861

**Fig 2:**
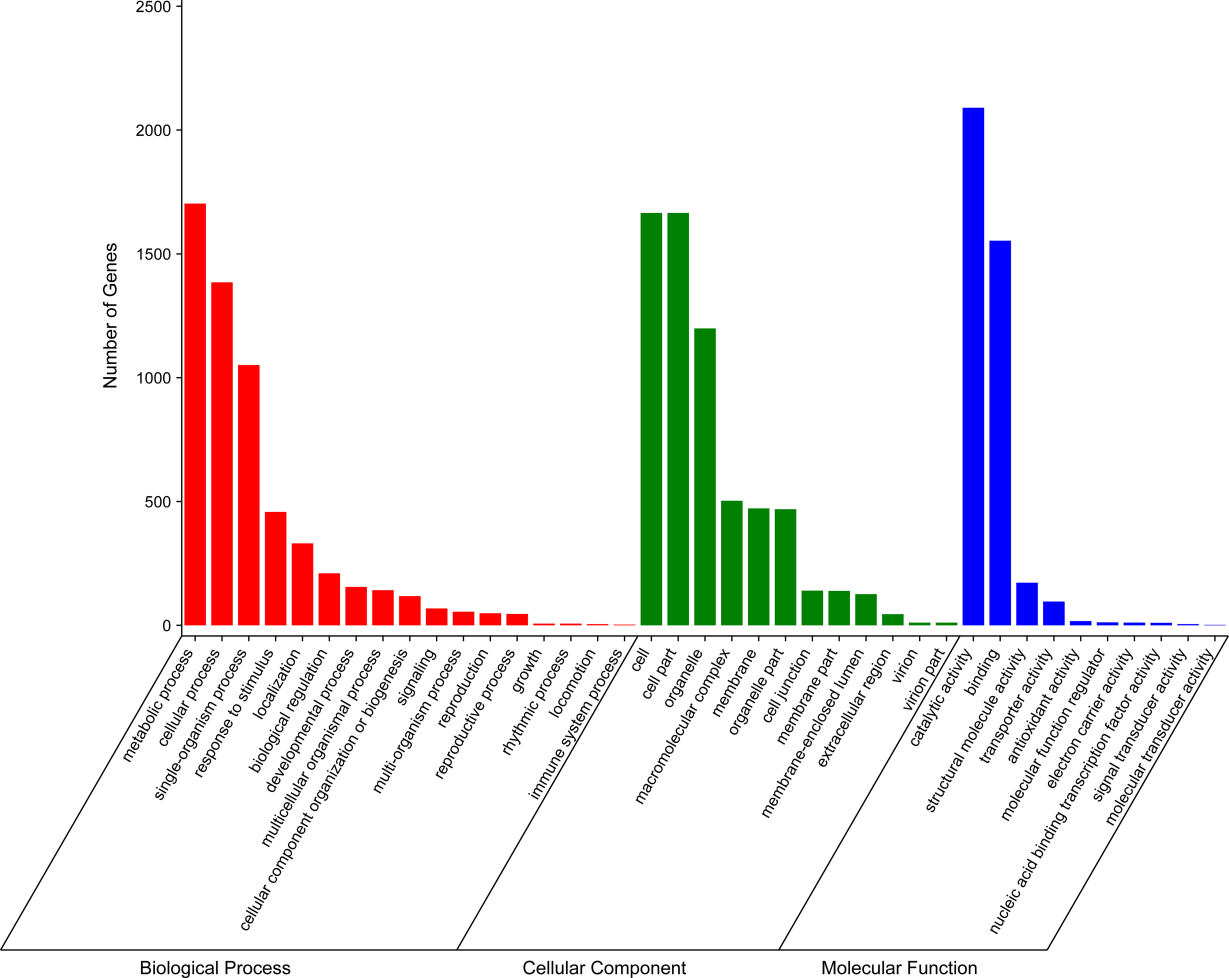
Gene Ontology (GO) classification of assembled unigenes mapped from contigs of the *P*. *haitanensis* transcriptome.

**Fig 3:**
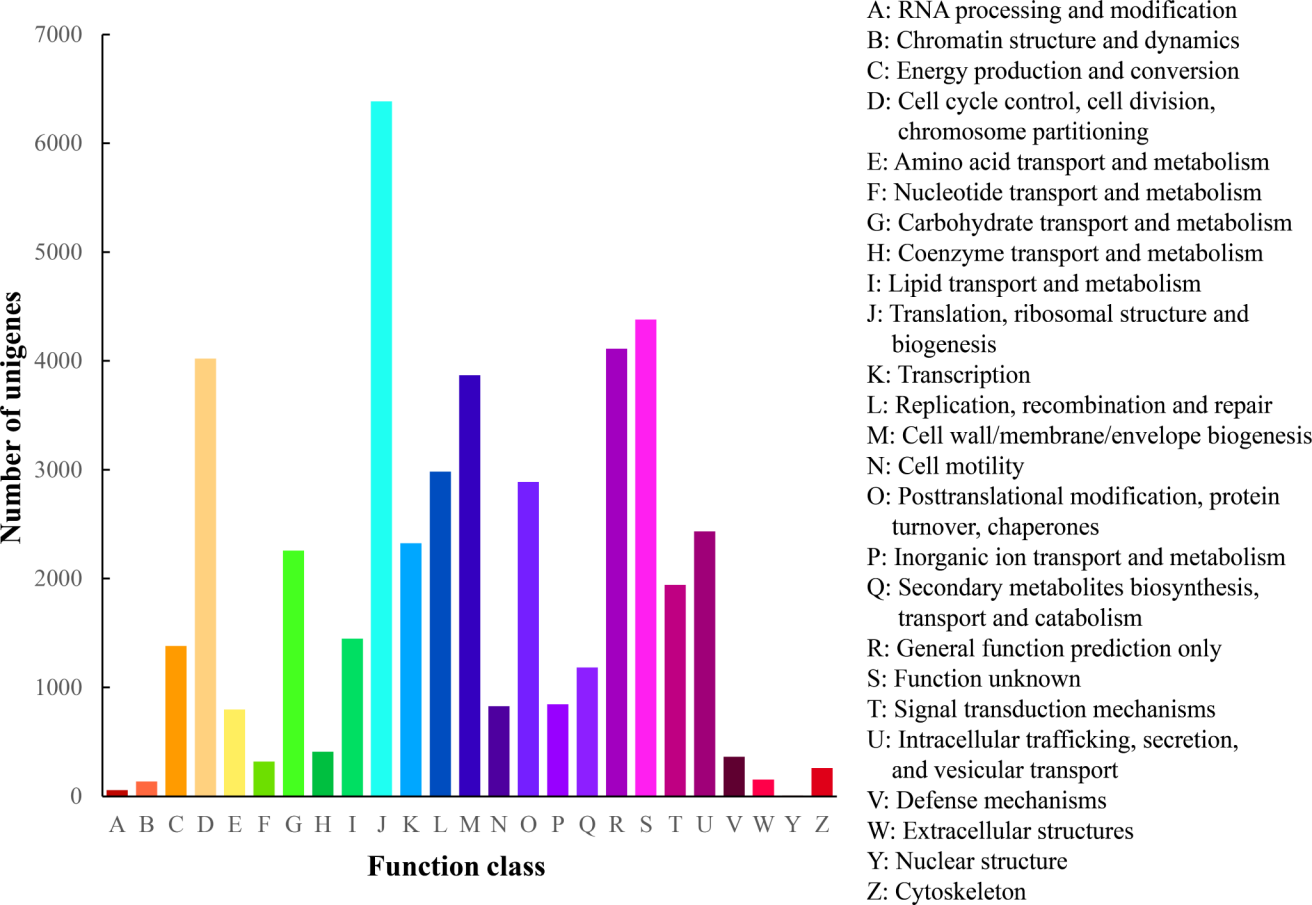
Histogram presentation of clusters of orthologous groups (COG) classification of assembled unigenes mapped from contigs of the *P*. *haitanensis* transcriptome.

BLAST analysis indicated that 23,074 (66.95%) of the 34,465 unigenes showed significant similarities to known proteins in the Nr database, and 20,682 (60.00%) had BLAST hits in the Swiss-Prot database (S2 File). These results might be due to a lack of genome information in *P*. *haitanensis*. Second, 3,824 sequences can be categorized into 41 GO functional groups (S2 File) based on sequence homology. In each of the three main categories (biological process, cellular component, and molecular function) of the GO classification, the terms “metabolic process”, “cell”, and ‘‘catalytic activity” were predominant, respectively. We also noticed a high-percentage of genes (11.04%) from categories of “Response to stimulus” (Fig 2). Third, a total of 13,953 unigenes were assigned to the COG categories (S2 File), which could predict and classify possible functions. Among the 25 COG categories, the cluster for “Translation, ribosomal structure, and biogenesis” represented the largest group (6,386; 45.77%), followed by “Function unknown” (4,378; 31.38%). We also detected 364 unigenes in the cluster for “Defense mechanism” (Fig 3). Fourth, to identify the active biological pathways in *P*. *haitanensis*, all unigenes were annotated with KEGG. In total, 8,124 (23.57%) of the 34,465 unigenes had significant matches in the database and were assigned to 122 KEGG pathways (S3 File). The pathways enriched by unigenes were the metabolic pathway (2,069 unigenes), RNA transport (1,015 unigenes), and the biosynthesis of secondary metabolites pathway (3,375 unigenes). Furthermore, some important pathways related to plant growth, development, and response to stress stimuli, including oxidative phosphorylation (320 unigenes), glycolysis/Gluconeogenesis (211), ubiquitin-mediated proteolysis (126 unigenes), carbon fixation in photosynthetic organisms (118 unigenes), peroxisome (109 unigenes), glutathione metabolism (86 unigenes), phosphatidylinositol signaling system (55 unigenes), and plant hormone signal transduction (14 unigenes) have also been successfully annotated (S3 File).

### Evaluation the reproducibility

**Fig 4:**
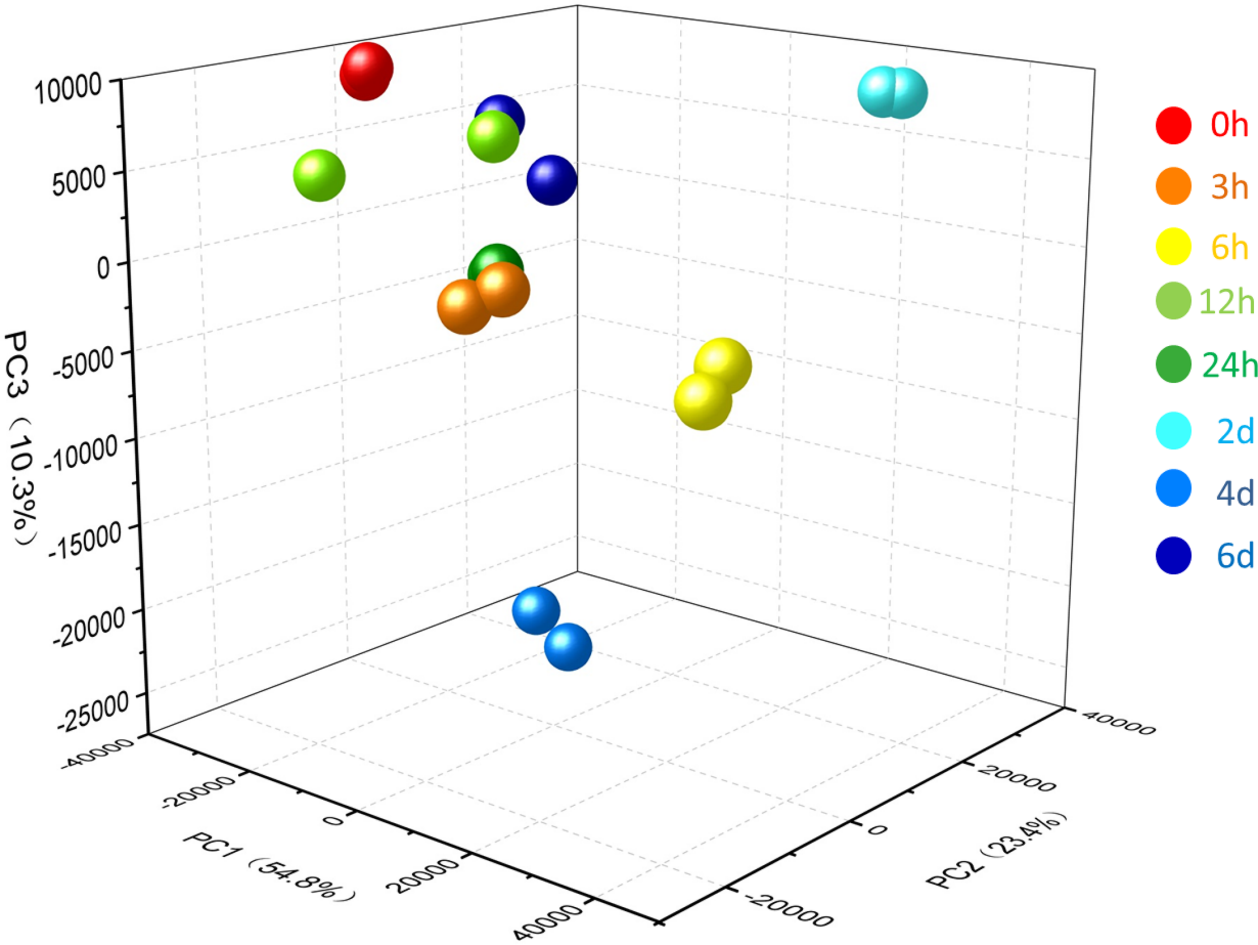
Principal component analysis of expression patterns of the 34,465 unigenes in each high temperature stress treatment. Numbers in parentheses represent the percentage of total variance explained by the first and second PC. Balls of the same color represent two biological replicates of each treatment.

To assess the reproducibility of the transcriptomic data, Pearson correlation analysis was performed on each two replicates of every high temperature treatment. The results showed that the Pearson correlation coefficient (r) between two replicate samples in each treatment was > 0.96, except for HT12h1 and HT12h2 (S1 Fig). As a first approach in the analysis of the complexity of the gene expression dataset, PCA could effectively retain the original information during comparisons of different samples. Fig 4 shows the the expression patterns in samples of different treatments were distinct, thereby suggesting that the expression patterns differed among various treatments using high-temperature stress.

### Differential expression analysis

The unigenes that were up- or down-regulated under high temperature treatments compared to the controls (0 h) (log_2_|fold change|≥ 1 and FDR≤ 0.001) were determined as high temperature-responsive genes. A total of 14,164 unigenes showed significantly different expression in at least one time point by high-temperature stresses (S4 File), which represented 41.10% of the total unigenes.

**Fig 5:**
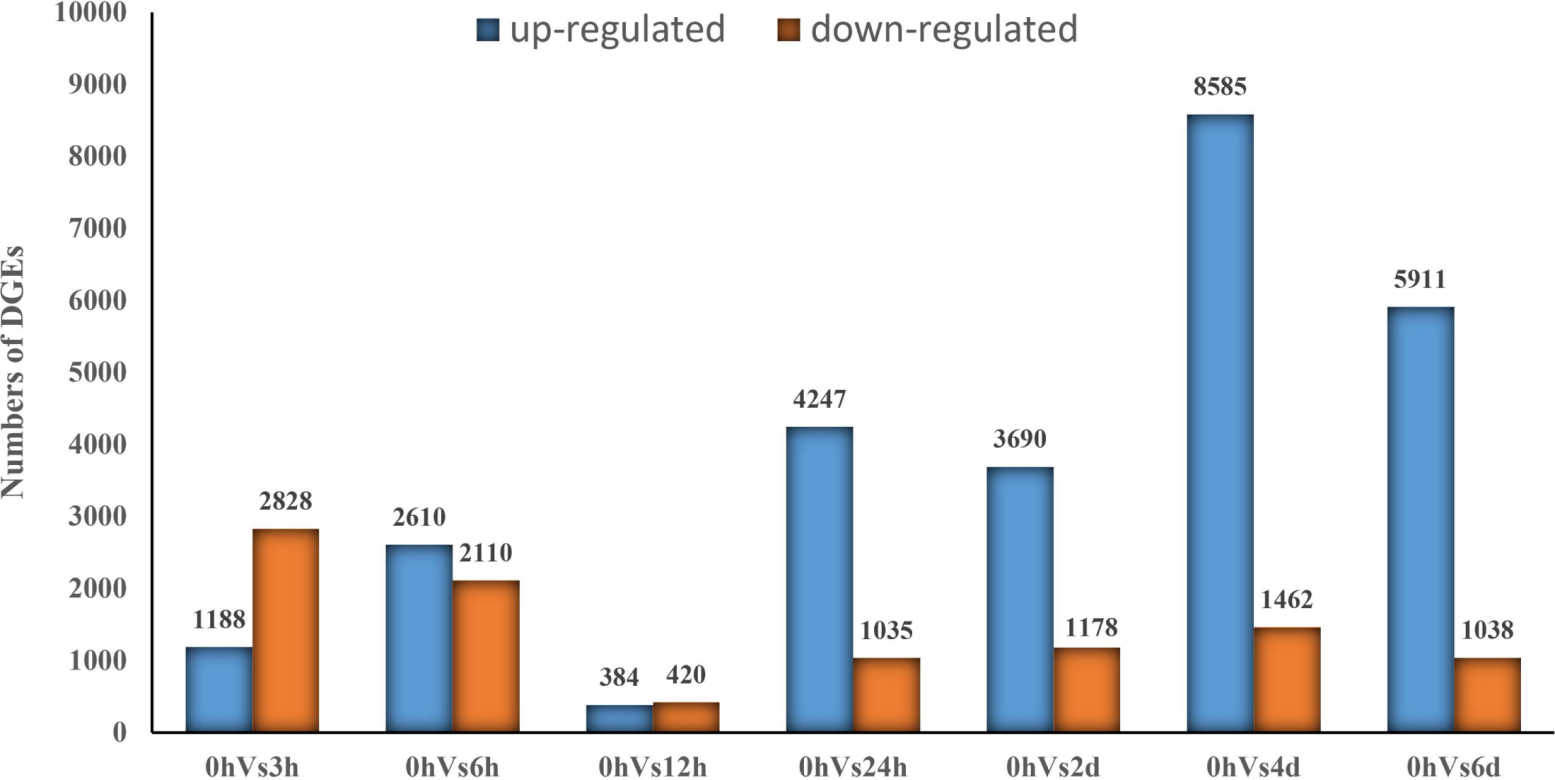
Numbers of differentially expressed unigenes in each comparison. The numbers on each column show the quantity of up- (blue) and down- (yellow) regulated unigenes.

Compared to the controls (0 h), under 3 h, 6 h, 12 h, 24 h, 2 d, 4 d, and 6 d of high temperature stress, 1,188, 2,610, 384, 4,247, 3,690, 8,585, and 5,911 unigenes were upregulated, and 2,828, 2,110, 420, 1,035, 1,178, 1,462, and 1,038 unigenes were downregulated, respectively (Fig 5). Among these, the number (804) of DEGs between 12 h and 0 h was the lowest, which indicated gene expression levels at 12 h of high temperature stress was close to that at normal condition (0 h), as suggested by a series of short-time physiological regulation responses to SHS. Then, over time, these gradually reached the high temperature tolerance point of *P*. *haitanensis*, which in turn activated long-time physiological regulation responses to SHS. These results also suggest that several early high temperature-responsive genes such as DEGs annotated by energy metabolism-related proteins, antioxidant defense system-related proteins, heat shock proteins (HSPs), signal transduction factors were turned off during the 12 h exposure to high temperature stress and then turned on again in response to long-term high temperature stress (LHS) after 12 h (S3 and S4 Files).

### Verification the gene expression profiles by qPCR

To further validate the expression levels of DEGs detected by RNA-seq technology, qPCR analysis was performed on 12 randomly selected DEGs. The results showed the R^2^ between the gene expression patterns detected by RNA sequencing and qPCR was 0.93, suggesting that the expression patterns detected by qPCR were in good agreement with those detected by RNA sequencing and thus valid (S2 Fig).

## Discussion

### *De novo* transcriptome sequencing and assembly of *P*. *haitanensis*

Global warming is predicted to be detrimental for plant growth and development. The temperature of the oceans has increased by 0.74°C during the 20^th^ century, and will continue to warm up in the 21st century [18]. Meanwhile, *P*. *haitanensis* farms often suffer from sustained higher temperatures than its optimal temperature for growing in early growth period (autumn in South China) [5, 6]. It is necessary, thus, to select and breed genetically thermoltolerant strains that can acclimate to global warming. However, the molecular mechanism underlying thermal tolerance of *P*. *haitanensis* remains largely unclear. In the present study, gene expression and regulation in response to short- and long-term temperature stresses (SHS and LHS) was investigated by performing genome-wide high-throughput transcriptomic sequencing for HTT. Wang et al. (2015) analyzed the transcriptome of *P*. *haitanensis* in response to osmotic stress, which identified a total of 28,536 unigenes with an average length and N50 size of 827 bp and 607 bp, respectively, after de novo assembly [19]. Using global transcriptome analysis of *P*. *haitanensis*, Xie et al. (2013) identified a total of 24,575 unigenes with a mean length of 645 bp and an N50 value of 913 bp [15]. This was indicative that the present assembly of the HTT strain of *P*. *haitanensis* transcriptome was reliable (Table 1). Homology matching against major public databases indicated that approximately one-third of the unigenes did not match with any known functions genes. This was probably owing to the genomic information of *Pyropia* is relatively highly limited in public databases.

Further, several DEGs in the present and previous studies on the responses of *Pyropia* to abiotic stresses were annotated to the categories of photosynthesis, energy metabolism, antioxidant defense system, and HSPs [10, 12, 19]. These findings indicate that different *Pyropia* species are capable of resisting to abiotic stresses by regulating common genes or pathways. However, HTT strain of *P*. *haitanensis* used in this study responded to sustained high temperature stress by extensively reprogramming its transcriptome instead of involving only a few specific stress responses-related genes or pathways.

### Overall changes in the transcriptome profile of *P*. *haitanensis* under different high temperature stresses

The results of the present study showed that almost 41% of the expressed genes had a modified expression pattern in at least one time point with different duration of high temperature treatment. This finding was in contrast to what has been observed in *Arabidopsis* [20], *Spinacia oleracea* [21], and *P*. *yezoensis* [12], where the ratio of DEGs generally ranges from 1% to 30%, and varies with different abiotic stresses, species, and the statistical treatment applied. Similarly, Dittami et al (2009) determined that almost 70% of the expressed genes were regulated by at least one of abiotic stresses examined in *Ectocarpus siliculosus* [22]. Wang et al. (2015) also determined that more than 50% of the expressed genes are up- or downregulated in the *Gracilaria lichenoides* transcriptome in response to osmotic and wounding stresses [23]. Therefore, our findings demonstrated that HTT of *P*. *haitanensis* responds to high-temperature stress by extensive transcriptome reprogramming.

Fig 5 shows that many unigenes of HTT rapidly responded to SHS, and acclimatory homeostasis was quickly established in its transcriptome within 12 h of its application. In contrast, the number of DEGs in the wild type at the different time points (within 24 h) during high temperature stress was obviously lower than in HTT, except at the 12 h time point [12]. It might due to the fact that blade cells of HTT are highly capable of rapidly adjusting their physiological activities to high temperatures. However, homeostasis was again disrupted, and a large number of unigenes exhibited different expression patterns during LHS (≥ 24 h). Kassahn et al. (2007) suggested that long-term exposure to high temperature stress in a coral reef fish (31°C for five days) induces most of the DEGs, which was in contrast to the results of short-term exposure treatment [24]. The number of upregulated genes was 3- to 6-fold higher than the number of downregulated genes when analyzing transcriptome of maize, wheat, and rice subjected to heat stresses [25–27]. Our data also showed that more genes were observed to be upregulated than downregulated during LHS in HTT (Fig 5). These results suggested that prolonged heat stress treatments induced increased expression of stress genes to repair damaged structures and to reduce potential damage. The differences in the responses to SHS and LHS might be due to the variations in the alterations in cellular redox balance [28].

### Photosynthesis-related unigenes in response to high temperature stress

Photosynthesis is one of the most sensitive systems that respond to high-temperature stress. Our previous study revealed *P*. *haitanensis* resisted high temperature by inhibiting photosynthesis [6], as reported in *P*. *yezoensis* [12]. In the present study, high temperature also appeared to have a negative impact on photosynthesis of HTT in *P*. *haitanensis* because most of the photosynthesis-related unigenes were downregulated (Table 2). For instance, several unigenes annotated as photosynthesis-antenna proteins were downregulated, especially under LHS, which resulted in the inability of the thallus of HTT to absorb sufficient light to activate photosynthesis. Furthermore, LHS might have inactivated *P*. *haitanensis* photosynthesis by the dissociation of relevant proteins, such as light-harvesting complex II [29]. However, the unigenes involved in photosynthesis clearly showed an upregulation after exposure to 12 h of heat stress in HTT, while it remained downregulated at the 12 h and 24 h time points of high temperature treatment in wild type of *P*. *haitanensis* [12]. Havaux (1993) demonstrated that potato leaves mediated thermotolerance of PSII when exposed to 2 h of high-temperature stress (35°C) [30]. In the present study, most unigenes of PSII were downregulated at the 3-h and 6-h time points of high temperature treatment, whereas all were upregulated at the 12-h time point (Table 2). Our data supported the hypothesis that acclamatory homeostasis is rapidly established in HTT of *P*. *haitanensis* transcriptome within a short time after exposure to SHS, including PSII thermotolerance.

**Table 2:** Enrichment of differentially expressed photosynthesis-related genes in various comparison groups. ‘+’ represents upregulation, and ‘-’ represents upregulation. The numbers between the parentheses represent the total number of upregulated and downregulated genes in the corresponding pathway, respectively.

Pathway		Number of unigenes (+/up- and -/downregulation)						
		0 VS_3h	0 VS_6h	0 VS_12h	0 VS_24h	0 VS_2d	0 VS_4d	0 VS_6d
**Photosynthesis-antenna proteins**		**(3+, 2-)**	**(4+, 10-)**	**(3+, 1-)**	**(3+, 8-)**	**(4+, 12-)**	**(13-)**	**(3+, 9-)**
*PC*		*2-*	*2+*,*2-*	*1+*	*1+*	*4-*	*3-*	*1+*, *2-*
*APC*			*2+*,*1-*	*1+*	*1+*,*1-*	*3+*,*3-*	*1-*	*1+*, *1-*
*LHC*		*3+*,*2-*	*3+*,*7-*	*3+*,*2-*	*3+*,*7-*	*4+*,*7-*	*9-*	*1+*, *7-*
**Photosynthesis**		**(6+, 13-)**	**(12+,13-)**	**(7+)**	**(13+, 7-)**	**(3+, 11-)**	**(14+, 12-)**	**(10+, 7-)**
	*PSI*	*1+*	*4+*,*1-*	*3+*	*4+*		*3+*,*1-*	*3+*,*1-*
	*PSII*	*3+*, *4-*	*5+*,*6-*	*4+*	*5+*,*3-*	*3+*,*5-*	*2+*,*6-*	*4+*,*4-*
	*Cytochrome b6-f complex iron-sulfur subunit*	*1-*	*1+*,*1-*		*1+*,*1-*		*2+*,*1-*	*1+*
	*Ferredoxin-NADP+ reductase*	*3-*	*2-*		*1+*,*1-*	*2-*	*1+*,*1-*	
	*F-type H+-transporting ATPase subunit gamma*	*2+*,*6-*	*2+*,*4-*		*2+*,*3-*	*5-*	*6+*,*4-*	*2+*,*2-*
**Porphyrin and chlorophyll metabolism**		**(2+, 20-)**	**(8+, 20-)**	**(2+)**	**(11+, 7-)**	**(8+, 8-)**	**(16+, 13-)**	**(4+, 10-)**
*Chlorophyll synthase*		*2-*						
*Protochlorophyllide reductase*		*2-*	*2-*		*2-*		*2-*	*1-*
*Magnesium-protoporphyrin O-methyltransferase*		*1-*	*1-*					*1-*
*Magnesium chelatase subunit D/H*		*2-/1+*	*2-/5+*,*2-*		*0/6+*	*1-/5+*,*1-*	*2-/2-*,*7+*	*0/1+*
*Light-independent protochlorophyllide reductase subunit N/L*			*2-/2-*					*1+/1+*
*Magnesium-protoporphyrin IX monomethyl ester (oxidative) cyclase*				*1+*	*1+*			*1+*
*5-Aminolevulinate dehydratase*		*1-*	*1-*					
*Hydroxymethylbilane synthase*		*1-*	*1-*					
*Uroporphyrinogen-III synthase*		*1-*	*1-*			*1-*		
*Uroporphyrinogen decarboxylase*		*1-*	*1-*			*1-*	*1-*	*1-*
*Coproporphyrinogen III oxidase*		*1-*	*1-*		*1-*	*1-*	*1-*	*1-*
*Oxygen-dependent protoporphyrinogen oxidase*		*1-*	*1-*		*1-*		*1+*	
*Others*		*1+*,*13-*	*3+*,*3-*	*1+*	*4+*,*3-*	*3+*,*3-*	*8+*,*5-*	*6-*

Photosynthetic adaption to high temperature involves in improving heat stability of the photosynthetic apparatus. Although photosynthetic electron transport in *Pisum sativum* leaves started to decline at high-temperature conditions of above 35°C, which subsequently resulted in an improvement in the thermal stability of the thylakoid membranes [31]. The majority of unigenes encoding PSI reaction center subunits were upregulated by high temperature stress, but most unigenes related to PSII were downregulated except at the 12-h time point (Table 2), indicating that PSI is more stable than PS II in the presence of higher temperatures stress. This finding is consistent with those of previous studies. Gao et al. (2013) revealed that the cyclic flow around PS I in *P*. *haitanensis* was still active, whereas the linear electron flow in PSII was inactive due to severe desiccation [32]. Lu et al. (2016) also determined that the effective photochemical quantum yield of PS I of *P*. *yezoensis* remained high, whereas that of PS II was almost depleted under high salt stress, which might in part be due to the enhancement of the cyclic electron flow around PS I by NADPH derived from the pentose phosphate pathway (PPP) [33]. Here, the unigenes related to the PPP appeared to be significantly upregulated with sustained high-temperature conditions (Table 3). Therefore, we also suggested that the PPP provided NADPH that was utilized in the cyclic electron flow around PS I because the activity of PS II decreased as HTT of *P*. *haitanensis* adapted to heat stress.

**Table 3:** Differentially expressed phosphatidylinositol and brassinosteroids-related gene enrichment in different comparison groups. ‘+’ represents upregulation, and ‘-’ represents upregulation. The numbers between the parentheses represent the total number of upregulated and downregulated genes in the corresponding pathway, respectively.

Pathway		Numbers of unigenes (+/up-, -/downregulation)						
		0_VS_3h	0_VS_6h	0_VS_12h	0_VS_24h	0_VS_2d	0VS_4d	0VS_6d
**Phosphatidylinositol signaling system**		**(1-)**	**(3+)**	**(1+)**	**(7+)**	**(7+)**	**(22+,2-)**	**(13+)**
	*PI synthesis*	1-			1+	1+	1+	2+
	*PIPK*				1+	1+	1+	2+
	*PI4K*					1+	2+	
	*PLC*		1+		2+		7+	2+
	*PIK3C3*					1+	1+	
	*IMPA*				1+	1+	1+	1+
	*VTC4*				1+	1+	1+	1+
	*CDS1*		1+	1+	1+	1+	3+	1+
	*Calm*				2+	2+	7+,2-	8+
**Brassinosteroids synhesis**		**(1+, 3-)**	**(2+)**	**(0)**	**(6+)**	**(4+)**	**(9+)**	**(10+)**
	*SMT1*							2+
	*CYP51G1*	2-					1+	2+
	*SMO2*				2+	2+	2+	1+
	*STE1*				1+	1+	2+	3+
	*DWF1*						2+	2+
	*DWF5*	1+	1+				1+	
	*Cycloartenol synthase*		1+		1+	1+	1+	
	*CPI1*	1-			1+			
	*FK*				1+			

Chlorophyll (Chl) biosynthesis in plants decreases upon exposure to high-temperature stress [34]. The activity of 5-aminolevulinate dehydratase (ALAD), the first enzyme of the pyrrole biosynthetic pathway, decreased with high-temperature treatment [35, 36]. Yamasaki (2002) have also reported that Chl biosynthesis in winter wheat decreased by about 30% with 35°C high temperature treatment compared to that using a control temperature of 25°C [37]. Yordanov (1986) demonstrated an inhibition of Chl biosynthesis in etiolated barley seedlings after high-temperature treatment for 4 and 8 h, which resulted from a decrease in the number or extent of protochlorophyllide transformation [38]. In the present study, porphyrin and chlorophyll metabolism were successfully annotated, and the majority of the DEGs in this pathway exhibited downregulation with high temperature stress, including ALAD and protochlorophyllide reductase (Table 2). In addition, protochlorophyllide synthesis, porphobilinogen deaminase, and chlorophyll synthase were analogously affected. These results suggested that high temperature stress results in the reduction of Chl biosynthesis in HTT of *P*. *haitanensis*, which in turn was advantageous in HTT because the accumulation of toxic ROS decreased [19].

### Energy metabolism-related genes in response to high temperature stress

**Fig 6:**
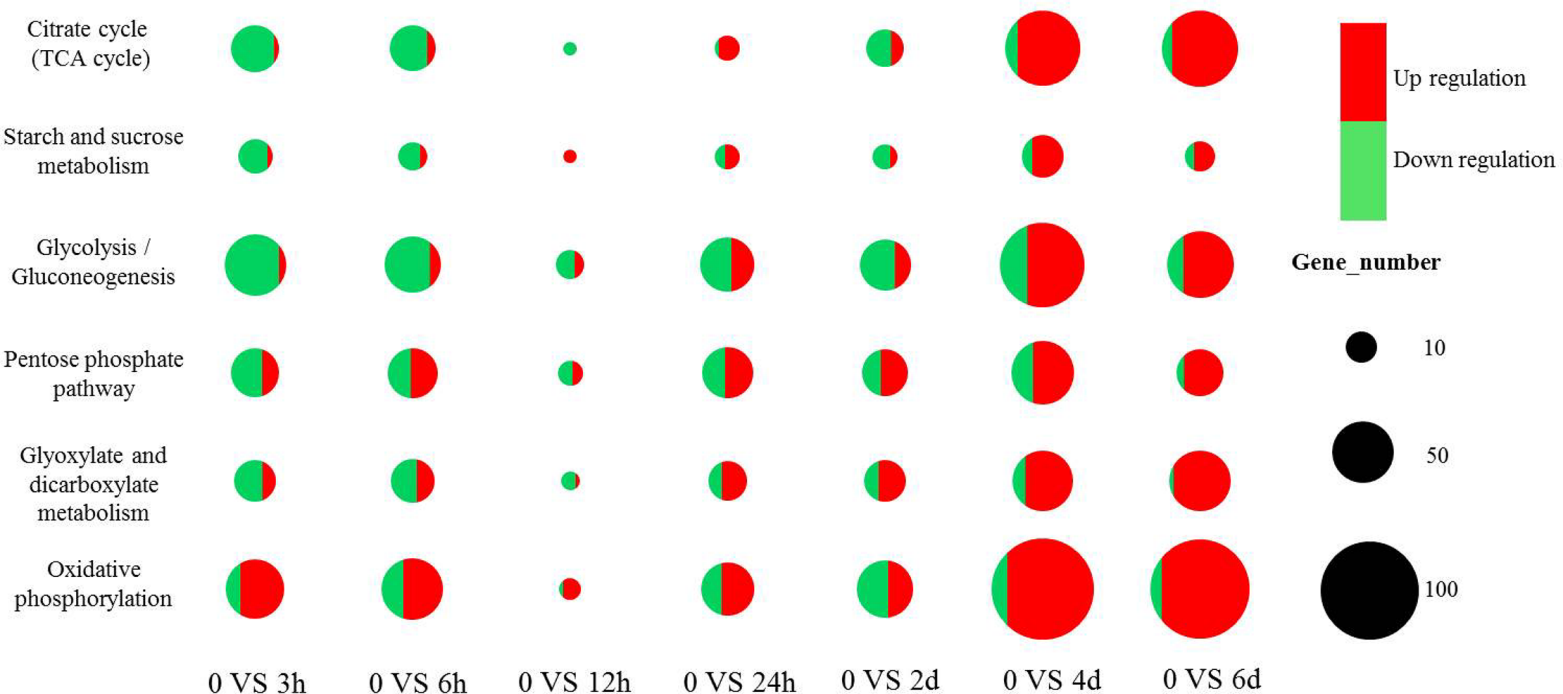
The statistics diagram of differently expressed genes relate to energy metabolisms at different time point under high-temperature stress.

Plants often drive a network of energy metabolism events in response to abiotic stresses [39, 40]. In the current study, the majority of DGEs related to energy metabolism were downregulated during SHS, including oxidative phosphorylation, glyoxylate and dicarboxylate metabolism, citrate cycle (TCA cycle), PPP, glycolysis/gluconeogenesis, and starch and sucrose metabolism (Fig 6). High temperature induced a downregulation of corresponding genes to modulate activities such as starch accumulation, carbohydrate metabolism, and sucrose synthesis [41]. Similarly, Xu et al. (2014, 2016) pointed out that the blades of *P*. *haitanensis* can avoid cell damage under stress by reducing unnecessary energy consumption [4, 6]. However, when stress levels excessively deteriorate, the corresponding energy shortage in plants would result in an enhancement of inherent pathways of carbohydrate metabolism and the induction of alternative pathways such as glycolysis, in order to provide energy and the related carbon skeletons for key metabolic processes [42]. In the present study, LHS induced the striking upregulation of unigenes of energy metabolism. For instance, the accumulation of sugar phosphates involved in glycolysis and PPP indicated the flow of carbon from glycolysis into the PPP, possibly to supply NADPH for antioxidation [42]. In addition, we also screened more than 50 upregulated unigenes that were annotated to the TCA cycle in both the 4-d and 6-d high temperature treatments. The TCA cycle is responsible for the oxidation of respiratory substrates that drive ATP synthesis [43] and thus plays a significant role in a plant’s capacity to resist high temperature and other abiotic stresses [44]. Therefore, the results of the present study suggest that the TCA cycle might also play a crucial role in the adaptation of HTT of *P*. *haitanensis* to heat stress.

### Antioxidant-related unigenes that respond to high-temperature stress

Heat stress can induce the overproduction of reactive oxygen species (ROS) in the chloroplastic PSII reaction center [45]. Although ROS acts as second messengers in response to abiotic stress [23], it would impair intracellular organelles and the physiological processes involved in plant growth and development when plants could not effectively scavenge redundant ROS. Plant tolerance to high temperatures seems to be associated with an increase in the activity of antioxidant enzymes [46, 47].

**Fig 7:**
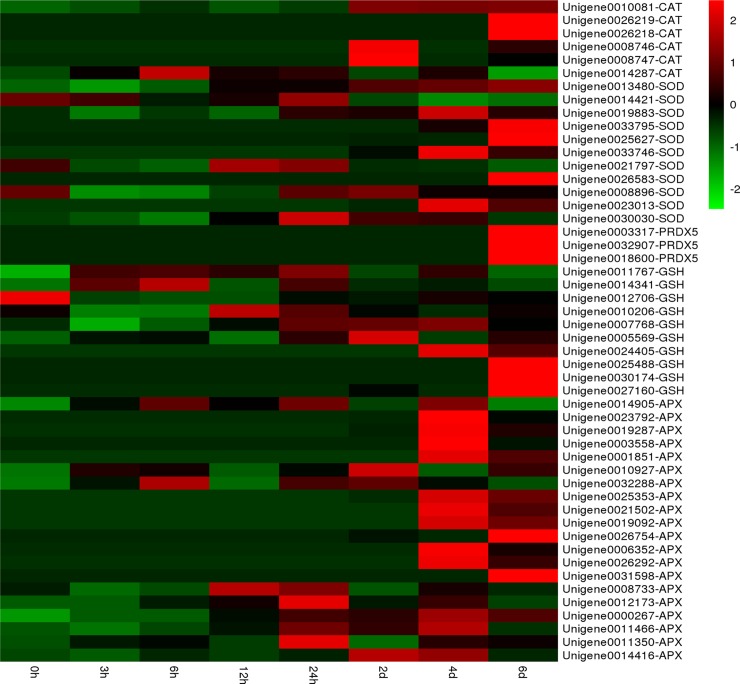
Expression patterns of antioxidant-related unigenes at different time points during exposure to high-temperature stress via hierarchical clustering. Red: upregulation; Green: downregulation.

In the present study, the expression of catalase (CAT) unigenes, a hydrogen peroxide scavenger enzyme, showed gradual upregulation with sustained high temperature (Fig 7). The sudden enhancement of antioxidant systems after SHS presumably allows the cells to prevent the establishment of a severe oxidative stress. This is also consistent with acclimatory homeostasis that rapidly established in the transcriptome within 12 h of application of high temperature stress. Notably, the respective fold-change of two CAT unigenes was 9- or 11-fold higher at the 2-d or 6-d time points (Fig 7). Meanwhile, the activity of superoxide dismutases (SOD), which is involved in high-temperature tolerance in *P*. *haitanensis* [48], was also induced by prolonged exposure (4 d and 6 d) to high temperature conditions. Two and five SOD unigenes showed a 10-fold upregulation with 4 d and 6 d of sustained heat stress, respectively. Furthermore, three peroxiredoxin 5 (PRDX5) unigenes exhibited a 10-fold upregulation only after 6 d of exposure to 29°C. We also observed that unigenes related to ascorbate and aldarate metabolism were significantly upregulated during high temperature treatment, especially in LHS. These results demonstrate that the prolonged exposure to heat stress could lead to more severe oxidative stress than short-term exposure, and antioxidant systems play a crucial role in the response of HTT of *P*. *haitanensis* to heat stress.

### Ubiquitin mediates proteolysis system-related genes in response to high-temperature stress

**Fig 8:**
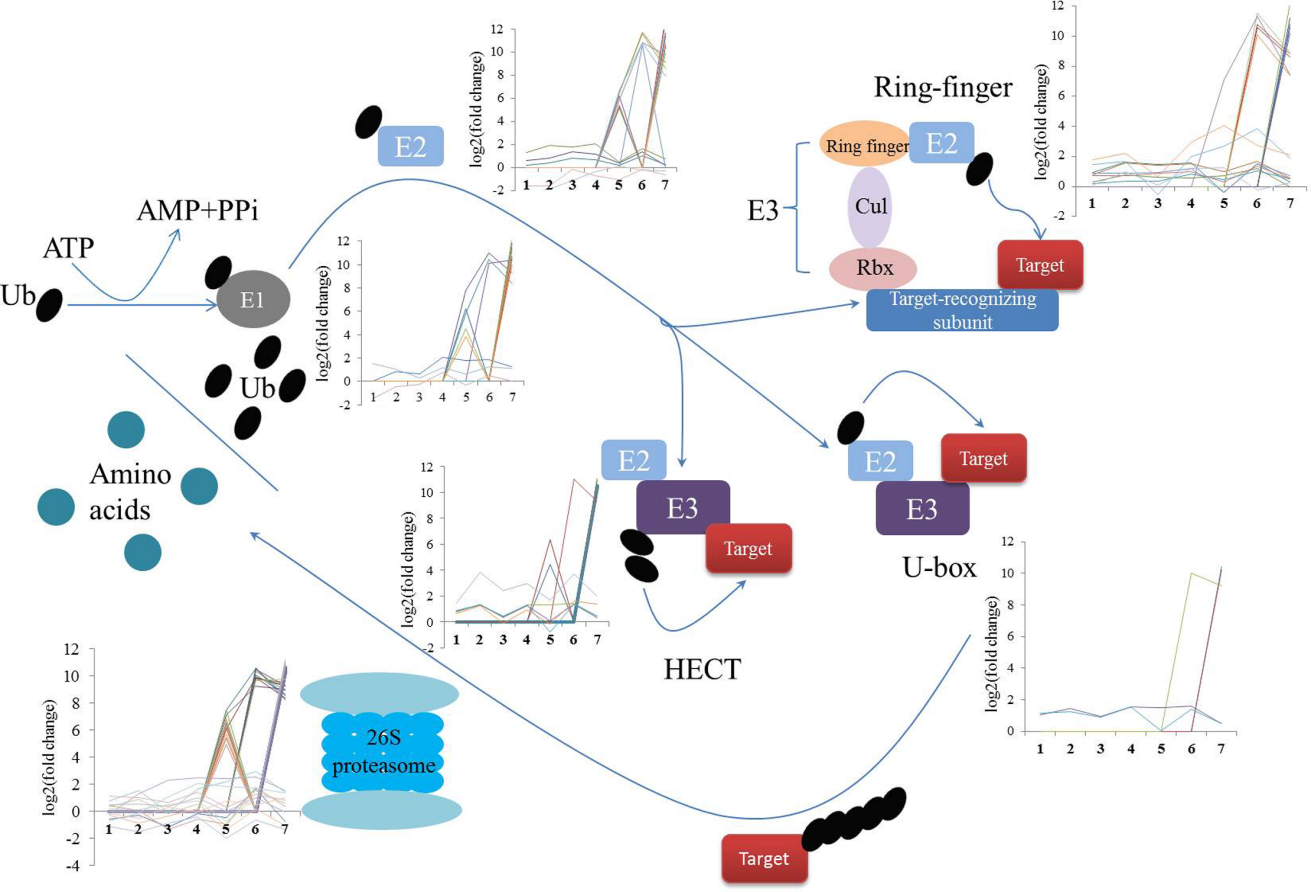
Expression profiles of differentially expressed genes involved in ubiquitin-mediated proteolysis in the *P*. *haitanensis* transcriptome. 1, 2, 3, 4, 5, 6, 7 in the X-axis of different expression diagram represent 0_VS_3h, 0_VS_6h, 0_VS_12h, 0_VS_24h, 0_VS_2d, 0_VS_4d, 0_VS_6d.

Genes involved in the ubiquitin-mediated proteolysis pathway are considered as key transcription factors that respond to abiotic stresses [49]. The process of attachment to ubiquitin to target proteins depends on three enzymes: ubiquitin activating enzyme (E1), ubiquitin conjugating enzyme (E2), and ubiquitin ligase (E3). The target proteins linked to ubiquitin are then degraded by the 26S proteasome. Recently, transcriptome analyses have revealed that hundreds of ubiquitination-related genes exhibited different expression patterns when plants are subjected to abiotic stresses [50, 51]. Here, 126 unigenes involved in ubiquitin-mediated proteolysis have been annotated in the global description of HTT transcriptome (S4 File). Similarly, we have also found some unigenes of ubiquitin-mediated proteolysis that were upregulated during SHS (Fig 8). Huang and Xu (2008) also observed an enhancement of ubiquitin and conjugated-ubiquitin synthesis in mesquite and soybean during the first 30 min of exposure to heat stress [52]. These findings thus indicate that ubiquitin-mediated proteolysis plays a significant role in the development of high-temperature resistance in HTT.

Compared to SHS, the unigenes related to ubiquitin-mediated proteolysis, including E1, E2, three types of E3, and the SCF complex, all upregulated under LHS conditions (Fig 8). Meanwhile, the majority of genes involved in the 26S proteasome pathway were also upregulated under LHS conditions. This might be due to the fact that the *P*. *haitanensis* blades suffered from severe heat stresses with LHS, thereby leading to the ubiquitin-proteasome system functions in the nucleus and cytoplasm to remove damaged or misfolded proteins and to mediate the levels of regulatory proteins [49]. Several E3 ligases accelerate the responses of different plant species to abiotic stresses stimuli by modifying the major corresponding downstream transcription factors that are related to stresses [39, 53]. Among all types of E3 ligases, the class of really interesting new gene (RING)-type (RING finger) E3 ligases is crucial for plants to develop abiotic stress tolerance. Differential expression analysis showed that the activity of RING finger E3 ligases was induced by LHS, but not SHS. These findings suggest that ubiquitination played a central role in the regulation of the transcriptional changes required for HTT of *P*. *haitanensis* to adapt to high temperature stress.

### Signal transduction-related genes in response to high temperature stress

The phosphatidylinositol (PI) signaling system is crucial for cells to response to various environmental stimuli [54]. The activities of phospholipase D and phosphatidylinositolphosphate kinase (PIPK) were induced in tobacco cells treated with 40°C [55]. In addition, phospholipase C (PLC) modulated an oxidative-stress signal from the plasma membrane in response to heat shock [56]. In the current study, PI4K, PIPK, PLC, and other PI transcripts were annotated in the HTT transcriptome. There were 7, 7, 22, and 13 PI genes that showed marked upregulation in the comparison groups of 24 h/CK, 2 d/CK, 4 d/CK, and 6 d/CK, respectively, whereas the expression of PI genes did not significantly change within SHS. Furthermore, PI interacts with a calcium-related signaling network [57, 58]. Several calmodulin unigenes exhibited strikingly different expression patterns between LSH and CK (Table 3); thereby suggesting that PI signal transduction mediates the response of HTT to long-term heat stimuli.

Brassinosteroids (BRs), a group of naturally occurring novel steroidal hormones in plants, interact with ROS in various ways. BRs could serve an antioxidant function and suppress lipid peroxidation during abiotic stresses [59]. Thussagunpanit et al. (2014) revealed that BRs increased chlorophyll and carotenoid levels as well as the rate of carbon dioxide assimilation, resulting in the resistance of rice seedlings to high temperature [60]. Jin et al. (2015) reported that BRs could facilitate the activity of SOD and APX, and reduced glutathione levels in *Ficus concinna* seedlings to mitigate the oxidative stress caused by high temperature [61]. Here, all unigenes involved in BRs biosynthesis such as cycloartenol synthase, SMO2, and DWF1, significantly upregulated during LHS (Table 3). Furthermore, both the activity of antioxidant enzymes and PSI during LHS also increased (Fig 7, Table 2), thereby suggesting that BRs protects photosynthesis and the antioxidant system of *P*. *haitanensis* from high-temperature stress [60–62]. Hardtke et al. (2007) also observed that BRs signaling and biosynthesis might be linked to an emerging upstream connection that involves calcium-calmodulin and PI signaling [63]. These findings indicated a crosstalk between BRs and calcium, and PI plays an important role in the development of thermotolerance in HTT.

### HSPs and other chaperone genes that respond to high-temperature stress

**Fig 9:**
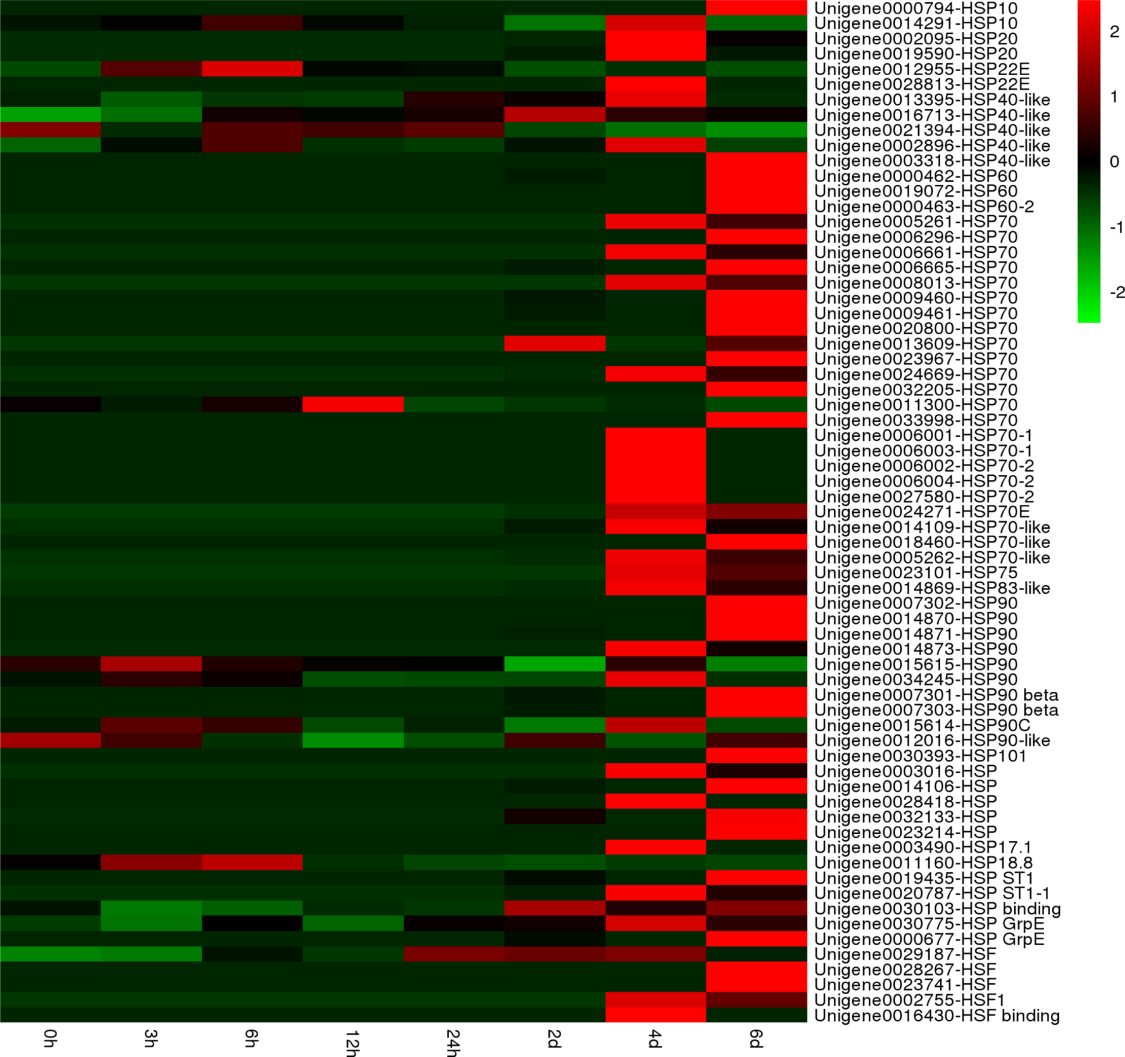
Heat map of significantly different expression genes encoding heat shock proteins (Hsps) under high temperature stress. Red: upregulation; Green: downregulation.

One adverse impact of high temperature stress on cells is the production and accumulation of denatured or misfolded proteins, which is cytotoxic due to the formation of protein aggregates [64]. HSPs and other chaperones play key roles in protein folding, stabilizing partially unfolded proteins, and eliminating non-native aggregations under various abiotic stresses. The high activity of HSPs is one of the common mechanisms underlying resistance to high temperature in various organisms [65–68]. Several studies have summarized that HSPs function as molecular chaperones that maintain protein folding homeostasis, which in turn is related to the establishment of thermotolerance [66, 69]. Coincident with this, 62 unigenes annotating various HSPs were upregulated in at least one time point during high-temperature treatment in the present study (Fig 9).

Among the five major families of HSPs/chaperones, the present study observed that the Hsp90 and Hsp70 families showed the most extensive upregulation (Fig 9), indicating that these mediated the response of HTT of *P*. *haitanensis* to high-temperature stress. Hsp70 could promote the proteolytic degradation of misfolded proteins by targeting the proteins to proteasomes or lysosomes [70]. A Hsp70 from *P*. *seriata* responds to high-temperature stress and involved in the establishment of thermotolerance [10]. Luo et al. (2014) found the mRNA levels of Hsp70 increased in *P*. *haitanensis* thalli exposed to 35°C [11]. Sun et al (2015) suggested that the Hsp70 mRNA maintain high expression levels prior to frequent habitat changes to allow *P*. *yezoensis* to acclimate to abiotic stresses [12]. In our previous study, we also obtained five full-length *P*. *haitanensis* Hsp70 genes using rapid amplification of complementary DNA ends, which were induced by a 29°C high temperature [14]. Here, 23 Hsp70 genes were mainly induced by LHS, indicating that it plays a significant role in prolong high-temperature resistance in HTT.

Hsp40 and GrpE, as Hsp70 co-chaperones, assist in a wide range of protein-folding processes have been also annotated in the *P*. *haitanensis* transcriptome (Fig 9). In addition, we also screened 10 Hsp90 genes that were differentially regulated during SHS and LHS (Fig 9). Similar to Hsp70, most Hsp90 genes were upregulation under LHS. The major role of Hsp90 is to manage protein folding, and it is also a key factor in signal transduction networks such as signaling kinases and steroid hormone receptors [71, 72]. Eight Hsp90 genes are upregulated when *Saccharina japonica* is exposed to heat stress [67]. Similarly, the expression of Hsp90 in some plants species is regulated by high temperatures such as *Arabidopsis* [73] and *Festuca arundinacea* [51]. Furthermore, small HSP (sHSP, ~17–30 kDa) and Hsp101, which were observed to be differentially expressed between SHS and LHS in HTT in the present study, are also essential in the establishment of thermotolerance in several plant species [74, 75]. sHSP could protect the oxygen evolving complex proteins of PSII and associate with thylakoids against heat stress [76]. Therefore, the results of the present study showed that some HSPs might have crucial effects on heat tolerance in HTT of *P*. *haitanensis*.

## Conclusions

**Fig 10:**
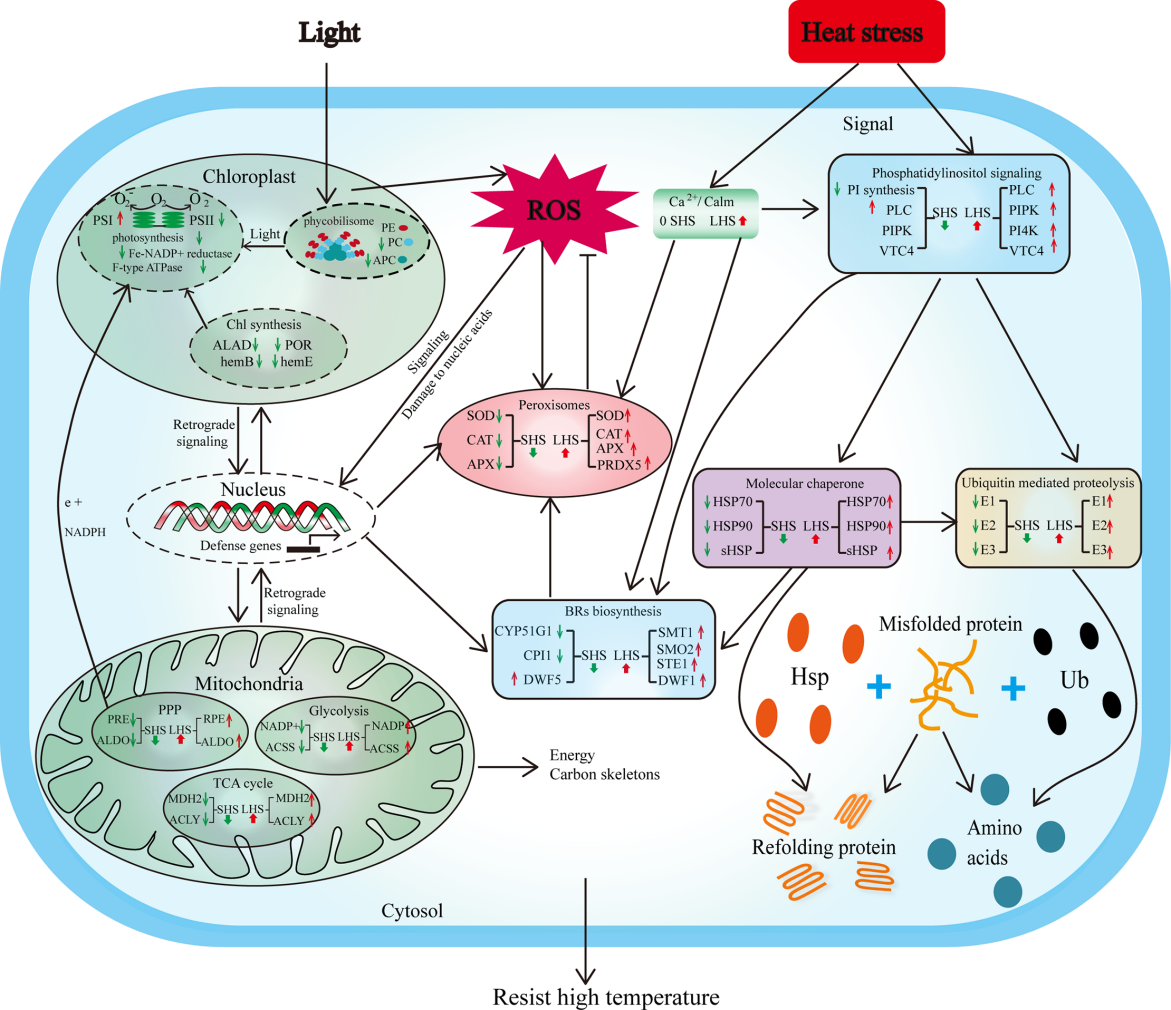
The crosstalk of different functional metabolic and signal transduction pathways that are involved in high temperature tolerance of *P*. *haitanensis*. Red arrows indicate upregulation, green arrows indicate downregulation.

In conclusion, the mechanism underlying the response of *P*. *haitanensis* to high temperature stresses, namely, SHS and LHS, was determined by performing genome-wide high-throughput transcriptomic sequencing of a high temperature-tolerant strain (HTT) for the first time. The effects of SHS and LHS on the mechanism of HTT to resist heat stress were relatively different. The initial response of HTT to SHS involved the inhibition of photosynthesis and energy metabolism to reduce unessential energy consumption, which was then followed by a rapid establishment of acclamatory homeostasis in the transcriptome within 12 h of application of high-temperature stress. On the other hand, LHS induced energy shortage, which led to an enhancement of the TCA cycle and other energy metabolism-related activities for the maintenance of energy and carbon skeletons, as well as antioxidant systems in order to resist severe oxidative stress. Meanwhile, signal transduction pathways such as that of PI, ubiquitin-mediated proteolysis, and Hsps also play a vital role in the response of HTT to high temperature, indicating the presence of a complex cross-talk network in response to high temperatures (Fig 10). The present study thus provides a better understanding of the mechanisms underlying high-temperature tolerance in *P*. *haitanensis* as well as a reference for improvement breeding schemes for high temperature-tolerant macroalgae that can adapt to current global warming conditions.

## Supporting information

S1 Table Information of the primers used in the qPCR analysis of *P*. *haitanensis* unigenes. (DOC)

S1 Fig Correlation tests for the replicates. The abscissa represents the value log_10_ (RPKM+1) of one duplicate; the ordinate represents the value log_10_ (RPKM+1) of the other duplicate. R is the Pearson Correlation Coefficient.
(DOC)

S2 Fig qPCR validation of RNA sequencing data on 12 selected genes (S1 File). (A) Expression values as detected by qPCR and RNA sequencing. (B) Correlation between qPCR and RNA sequencing analyses.
(DOC)

S1 File The quality of the assembly was analyzed by mapping the Illumina reads to the assembled transcriptome. (XLSX)

S2 File Functional annotation of 34,465 unigenes identified in the transcriptome of *P*. *haitanensis*. (XLS)

S3 File KEGG annotation of 34,465 unigenes identified in the transcriptome of *P*. *haitanensis*. (XLS)

S4 File Differentially expressed unigenes and their relative expression levels in each treatment. (XLS)
