# Supplementary material for: Transcriptomic study to understand thermal adaptation in a high temperature-tolerant strain of Pyropia haitanensis
Source: PLoS One. 2018 Apr 25;13(4):e0195842. doi: 10.1371/journal.pone.0195842 (PMC5919043; doi:10.1371/journal.pone.0195842)
Supplement: S1 Table — (DOC) [file pone.0195842.s001.doc]

**S1 Table: Information of the primers used in the qPCR analysis of *P. haitanensis* unigenes.**

| Gene ID | Primer sequences (5'→3') | Amplicon length(bp) | Amplification efficiency |
| --- | --- | --- | --- |
|  |  |  |  |
| Unigene0013193 | F: AGGGACTGCACCTTGGGG | 204 | 0.96 |
|  | R: GATTGAGATTGACTCGCTGTTTG |  |  |
| Unigene0010081 | F: CGCTTTAGCCTGGTCACGC | 103 | 1.04 |
|  | R: CCATATCCCAAATGCCCTCTG |  |  |
| Unigene0009343 | F: GCATCCAGAGCCTCATCCTG | 138 | 1.07 |
|  | R: TGGCGTAGACGACCGTATTG |  |  |
| Unigene0013975 | F: CAAGCGTTGCGTGGCGGTTGTGGAT | 177 | 1.03 |
|  | R: ACTCGGCAACCCTTCAGGTCCACTA |  |  |
| Unigene0011674 | F: TGCAGGGCTTCCAGGTGAC | 207 | 1.00 |
|  | R: CCGCATCCGCATTCTCCAC |  |  |
| Unigene0013820 | F: CGACGCTTAGGGCACAAC | 275 | 1.04 |
|  | R: AGCCCATTAGCAAATGTTCAG |  |  |
| Unigene0032440 | F: CGACGCTTAGGGCACAAC | 135 | 1.01 |
|  | R: TCAACTTGCCGTTCATCACT |  |  |
| Unigene0011300 | F: CAAGAAACACTACTATTCCGACAA | 160 | 0.98 |
|  | R: GGTGCAGGCATAATACCATCTA |  |  |
| Unigene0015244 | F: GATCCACCACATGCAGATTG | 158 | 1.02 |
|  | R: TGTGCACCTCCAGTACCTTG |  |  |
| Unigene0012955 | F: TTTCCCAGTCCTACCGCCTTCC | 132 | 1.03 |
|  | R: TCGCAATCGTCCGCTTCTCC |  |  |
| Unigene0011723 | F: CGACTACATTGCTACCCGCT | 181 | 0.94 |
|  | R: CACCTCCACAATCAACCGGA |  |  |
| Unigene0012119 | F: CCCGCCAAACTGTCTGCTCT | 316 | 1.04 |
|  | R: CGAGTGGCAGCAATCCGTAA |  |  |
| *PhUBC* | F: TCACAACGAGGATTTACCACC | 107 | 0.99 |
|  | R: GAGGAGCACCTTGGAAACG |  |  |
