# Supplementary material for: Transcriptomic study to understand thermal adaptation in a high temperature-tolerant strain of Pyropia haitanensis
Source: PLoS One. 2018 Apr 25;13(4):e0195842. doi: 10.1371/journal.pone.0195842 (PMC5919043; doi:10.1371/journal.pone.0195842)
Supplement: S1 Fig — The abscissa represents the value log10 (RPKM+1) of one duplicate; the ordinate represents the value log10 (RPKM+1) of the other duplicate. R is the Pearson Correlation Coefficient. (DOC) [file pone.0195842.s002.doc]

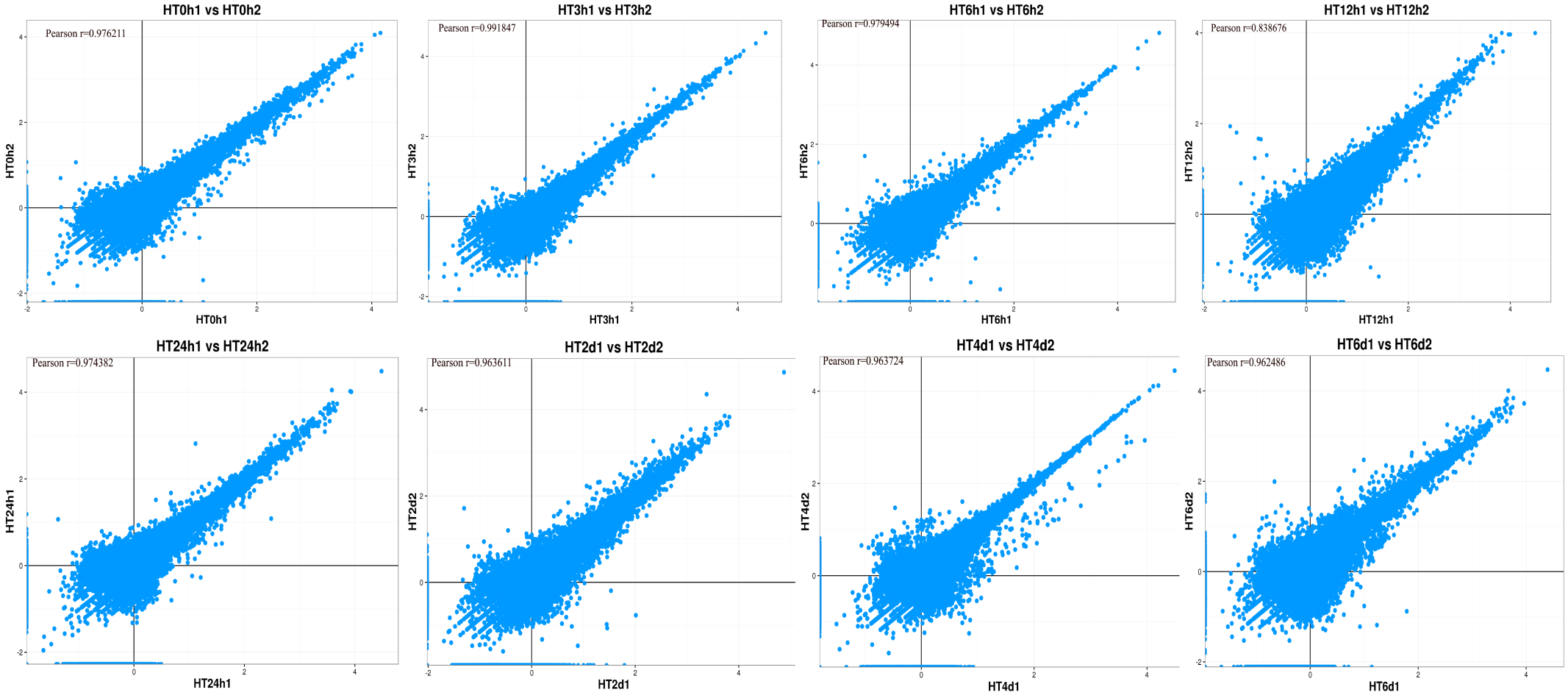


**S1 Fig:** Correlation tests for the replicates. The abscissa represents the value log_10_ (RPKM+1) of one duplicate; the ordinate represents the value log_10_ (RPKM+1) of the other duplicate. R is the Pearson Correlation Coefficient.
