# Supplementary material for: Transcriptomic study to understand thermal adaptation in a high temperature-tolerant strain of Pyropia haitanensis
Source: PLoS One. 2018 Apr 25;13(4):e0195842. doi: 10.1371/journal.pone.0195842 (PMC5919043; doi:10.1371/journal.pone.0195842)
Supplement: S2 Fig — (A) Expression values as detected by qPCR and RNA sequencing. (B) Correlation between qPCR and RNA sequencing analyses. (DOC) [file pone.0195842.s003.doc]

**S2 Fig:** qPCR validation of RNA sequencing data on 12 selected genes (S1 File). (A) Expression value detected by qPCR and RNA sequencing. (B) Correlation between qPCR and RNA-Sequencing.
